# Long‐term outcome of Coats' disease: Implications for the classification of foveal vascular pathologies

**DOI:** 10.1111/aos.17554

**Published:** 2025-07-05

**Authors:** Claudia Brockmann, Bert Müller, Shideh Schönfeld, Sibylle Winterhalter, Oliver Zeitz, Antonia M. Joussen

**Affiliations:** ^1^ Department of Ophthalmology Charité – Universitätsmedizin Berlin Berlin Germany; ^2^ Corporate Member of Freie Universität Berlin, Humboldt‐Universität Berlin Berlin Germany; ^3^ Berlin Institute of Health Berlin Germany; ^4^ Present address: Department of Ophthalmology Rostock University Medical Center Rostock Germany

**Keywords:** classification system Shields, Coats' disease, foveal vascular pathology, long‐term outcome

## Abstract

**Purpose:**

To investigate the long‐term outcome in patients with Coats' disease regarding initial stages.

**Methods:**

Within a retrospective, clinical, single centre study, patients with diagnosed Coats' disease treated from 1992 to 2019 (minimum follow‐up of 12 months) were investigated. A total of, 75 out of 123 patients met the inclusion criteria.

**Results:**

A total of, 77 eyes of 75 patients (80.5% male, 17.8 ± 18.6 years, median 10 years) were analysed. Mean follow‐up time was 7.2 ± 5.5 years (range 1.1–21.1 years, median 6.3 years). Initial and final visual acuity was lower the higher the disease stage was. The need for surgical interventions was higher in more advanced stages. Notably, within stage 2B, significant differences in final visual acuity were observed depending on the presence of foveal exudation only (0.20 ± 0.29 logMAR) as compared to eyes with foveal vascular abnormalities (1.20 ± 0.55 logMAR, *p* < 0.001).

**Conclusion:**

Based on our analyses, it is suggested to amend the well‐defined and established classification system according to Shields by sub‐dividing stage 2B (telangiectasia and exudation with foveal exudation) into 2B1 (telangiectasia and exudation with foveal exudation) and 2B2 (telangiectasia and exudation with foveal exudation and foveal Coats' typical vascular abnormalities), since both subgroups significantly differ with respect to prognosis of visual acuity.

## 1 INTRODUCTION

Coats' disease is a predominantly unilateral retinal vascular disease mainly in young male (Coats, 1908; Joussen et al., 2013). In 2000, Shields and Shields introduced a classification system (Shields et al., 2001), stratifying affected eyes into five major categories: stage 1, telangiectasia without any exudation; stage 2, telangiectasia with exudation, extrafoveal (2A) or foveal (2B); stage 3, exudative retinal detachment, subtotal and extrafoveal (3A1) or foveal (3A2), or total (3B); stage 4, total retinal detachment with secondary glaucoma; and stage 5, advanced end‐stage disease with total retinal detachment, often associated with cataract and progression to phthisis bulbi (Shields et al., 2001). Most recent investigations are based on this classification system (Al‐Qahtani et al., 2015; Brockmann et al., 2021; Dalvin et al., 2019; Khoo et al., 2019; Sen et al., 2019; Shields et al., 2019a, 2019b; Shields & Shields, 2002; Udyaver et al., 2019) and demonstrate considerable differences between stages regarding the course of visual acuity and the need for interventions. Furthermore, the necessity of repeated treatments, as well as the overall rate of globe preservation, correlates with stage (Sen et al., 2019; Shields et al., 2019a, 2019b; Udyaver et al., 2019). Nevertheless, in clinical practice, visual prognosis, especially for patients with central involvement, remains uncertain in many cases.

Affection of the posterior pole in Coats' disease was first described in 1912 by Leber. Leber's Miliary Aneurysms affect the macular area (Leber, 1912). While there are similarities to idiopathic juxtafoveal teleangiectasia, ectatic vessels are more widespread in Leber's Miliary aneurysems but remain associated with retinal vasculature. Central Leber's disease and Coats' disease were both considered to be one disease entity by Reese and were termed together as central Coats' disease from there on (Reese, 1956). Subsequently, differentiation between Coats' disease with foveal exudation originating from vascular abnormalities in the periphery and Coats' disease with foveal vascular abnormalities got gradually lost. Accordingly, Shields classification differentiates in stage 2 only between the presence or absence of foveal exudation but does not provide considerations regarding the location of vascular pathologies. Therefore, the present study hypothesizes that the exact localization of vascular abnormalities is a predictor for visual outcomes and the need for treatment.

## 2 METHODS

### 2.1 Patients data and ocular findings

A total of, 123 patients were examined and treated from 1992 up to 2019. Only patients with a minimum follow‐up of 12 months were included. A total of, 77 eyes of 75 patients were found to be eligible for inclusion. Data were retrospectively evaluated (demographic data, medical/ocular history, first diagnosis, symptoms, duration of symptoms, best corrected visual acuity (BCVA), intraocular pressure (IOP), slit lamp examination and fundus findings). Disease stages were classified according to Shields et al. (2001). Macular Coats' typical vascular abnormalities were defined as: telangiectasia, capillary bed abnormalities, distorted foveal avascular zone and microaneurysms. According to the ethical standards of the institutional and national research ethics committee and the Helsinki declaration (1964 and its later amendments), all patients consented.

### 2.2 Fundus imaging and fluorescein angiography

Fundus colour pictures were taken in mydriasis. If fluorescein angiography (FFA) was performed, intravenous sodium fluorescein (20%/3–5) was used and images were taken at all phases (early/middle/late). Before 2016, images were taken with the Zeiss FF450plus camera (Carl Zeiss Meditec/Jena/Deutschland) afterwards, predominantly as ultra‐widefield imaging (UWFI) and ultra‐widefield fluorescein angiography (UWFFA) using Optos California (Optos plc/Dunfermline/Scotland/UK). If the examination required general anaesthesia, fundus images including FFA were taken using RetCam® (Massie Research Laboratories Inc./Dublin/California).

### 2.3 Optical coherence tomography

Macular area was captured using Stratus‐OCT TM (Carl Zeiss Meditec/Jena/Germany) or spectral domain optical coherence tomography (SD‐OCT) SPECTRALIS® (Heidelberg Engineering GmbH/Heidelberg/Germany).

### 2.4 Data and imaging analyses

Decimal BCVA was converted to logarithm of minimum angle of resolution (logMAR) for statistical analysis. Averages are given as mean ± standard deviation (range minimum–maximum, median). Normally distributed variables with equal variances were compared using Student's *t*‐test; variables with unequal variances using Welch's two‐sample *t*‐test. Chi‐squared, Mann–Whitney *U* and McNemar tests were used for ordinal data. All statistical evaluations were accomplished using SPSS version 19.0 (IBM/Armonk/USA). A *p*‐value <0.05 was considered statistically significant.

## 3 RESULTS

### 3.1 Patients characteristics and stage distribution

**FIGURE 1:**
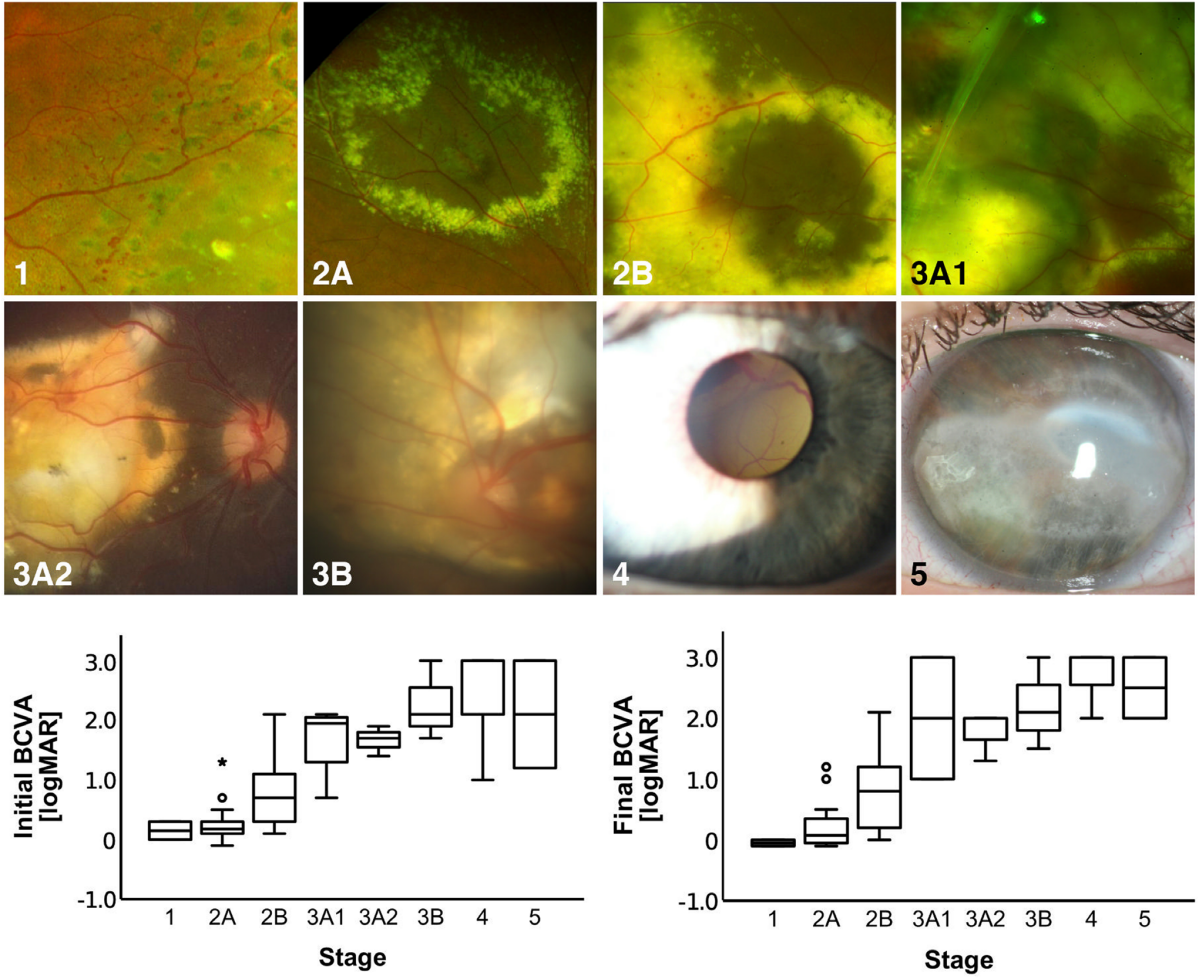
Upper row, exemplary representation of eyes of the study cohort with regard to the proposed stages of Coats' disease according to Shields. Stage 1, only telangiectasia; stage 2A, telangiectasia with extrafoveal exudation; stage 2B, telangiectasia with foveal exudation; stage 3A1, extrafoveal subtotal retinal detachment; stage 3A2, foveal subtotal retinal detachment; stage 3B, total retinal detachment; stage 4, total retinal detachment (retrolental) and secondary glaucoma with rubeosis iridis; stage 5, end‐stage disease with phthisis bulbi and band keratopathy. Right diagram, initial best corrected visual acuity (BCVA) given as logarithm of minimal angle of resolution (logMAR) with regard to the initial stage. Significances were omitted to avoid overload of the figure. Left diagram, final best corrected visual acuity (BCVA) given as logarithm of minimal angle of resolution (logMAR) with regard to the initial stage. Significances were omitted to avoid overload of the figure.

A total of, 77 eyes of 75 patients had a follow‐up time >12 months. Of these, 80.5% (61/75) were male, 19.5% (14/75) female. Mean age was 17.8 ± 18.6 years (range 1–71, median 10). Patients visited our department between April 1992 and September 2019. Follow‐up time was 7.2 ± 5.5 years (range 1.1–21.1, median 6.3). In 50.6% (39/77) right eye, in 49.4% (38/77) left eye were diagnosed with Coats' disease; two patients presented with bilateral affection. All stages were represented: 2.6% stage 1 (2/77), 20.8% 2A (16/77), 42.9% 2B (33/77), 11.7% 3A1 (9/77), 3.9% 3A2 (3/77), 6.5% 3B (5/77), 9.1% 4 (7/77) and 2.6% 5 (2/77). Exemplary fundus findings of each stage are shown in Figure 1 upper row.

### 3.2 Initial visual acuity and intraocular pressure

The overall BCVA at initial visit was 0.98 ± 0.83 logMAR (−1.0–3.0, median 0.90). From stage 1 to 5, there was an almost continuous decrease of BCVA as shown in Figure 1 left diagram and Table S1. While in stage 1 BCVA was 0.15 ± 0.21 logMAR, the mean BCVA of stage 2A was numerical—but not significantly—inferior (0.27 ± 0.34 logMAR; *p* = 0.648). Yet, compared to stage 1, the BCVA of stage 2B was not significantly different (*p* = 0.106). Stage 2B demonstrated a significant and clinically relevant decrease of BCVA, as compared to stage 2A (0.79 ± 0.54 logMAR; *p* < 0.001); however, with a wide range of initial BCVA. Stage 2B marks the transition of BCVA from initially good BCVA in early stages (1, 2A) to massive loss of BCVA in advanced stages. From stage 3A1 to 5, the mean BCVA was poor, ranging from 1.7 to 2.1 logMAR (3A1:1.68 ± 0.66; 3A2:1.67 ± 0.25; 3B:2.27 ± 0.67; 4:2.22 ± 0.74; 5:2.10 ± 1.27; Tables S1 and S2).

Overall IOP at initial visit measured 17.2 ± 9.0 mmHg. A significant raise of IOP was found in stage 4 (36.6 ± 16.7) compared to 2A (*p* = 0.036) and 3A1 (*p* = 0.017). A marginal *p*‐value was seen between stage 4 and 2B (*p* = 0.050); no differences between the mean IOPs were found between stage 4 and 3A2 (*p* = 0.304), 3B (*p* = 0.178) or 5 (*p* = 0.089), which might be attributed to a small number of eyes and a wide IOP range within these stages (Table S1).

### 3.3 Symptoms at initial visit

**FIGURE 2:**
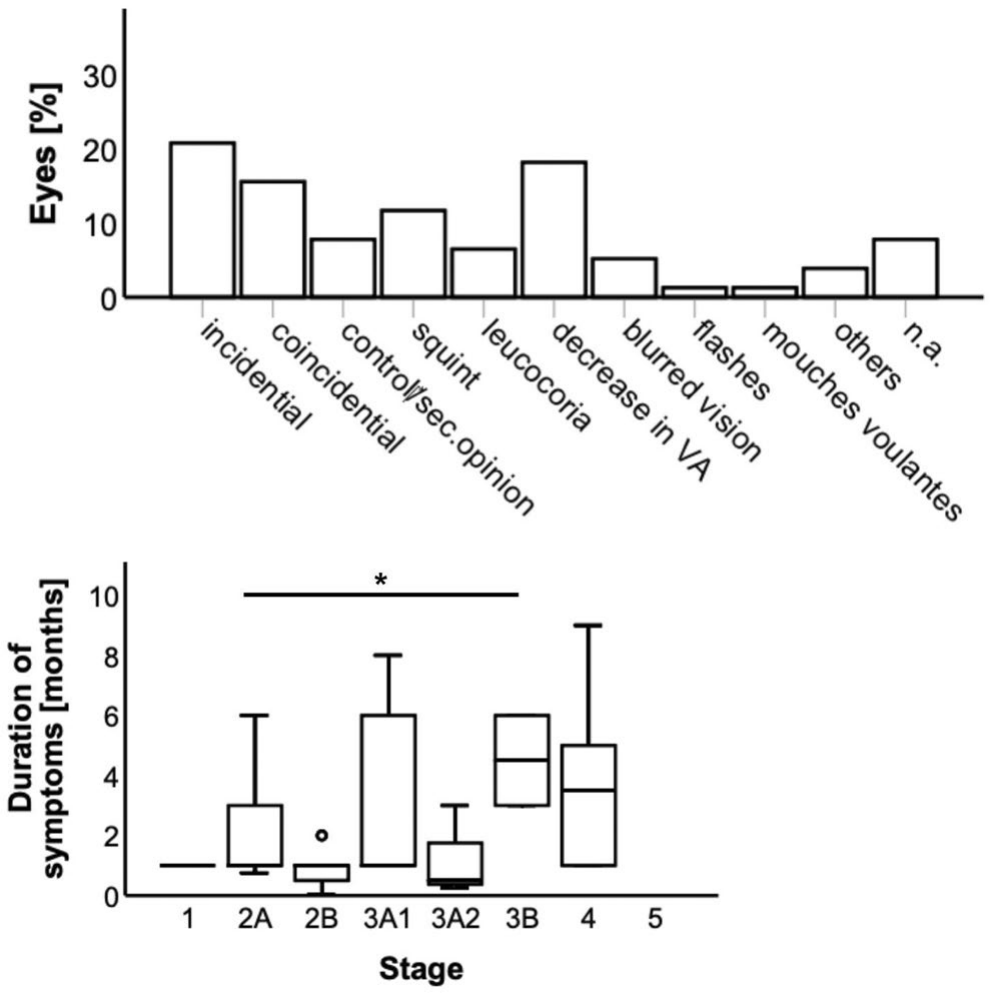
Symptoms across all stages (upper row) and duration of symptoms (lower row). Only reported symptoms less than 12 months were included. sec, second; VA, visual acuity; n.a., no answer; *, *p* < 0.05.

During the initial visit, reasons for presentation, in particular, first symptoms were asked (Figure 2 upper row). When including all stages, in 44.2% (34/77) diagnosis was incidentally (20.8%; 16/77), coincidental that means during examination of other ocular pathologies (15.6%; 12/77), or patient presented for a second opinion or follow‐up (7.8%; 6/77). Second category was appointment due to symptoms noticed by family members or friends (18.2%; 14/77) such as squint (11.7%; 9/77) or leukocoria (6.5%; 5/77). Subjective sensations were the first symptoms in 26.0% (20/77), such as decreased visual acuity (18.2%; 14/77), blurred vision (5.2%; 4/77), flashes (1.3%; 1/77) or mouches volantes (1.3%; 1/77). Three patients (3.9%) complained about unspecific symptoms such as headache. No specific data regarding symptoms at the initial visit was filed for six patients (7.8%).

Initial symptoms were compared between stages with the highest number of patients (2A/2B/3A1; Figure S1). While in stage 2A one‐third of cases (31.3%; 5/16) were diagnosed incidentally, most cases of stage 2B presented due to a decrease in visual acuity (27.3%; 9/33). In contrast, the most common reason for ophthalmic examination in stage 3A1 was squint (33.3%; 3/9).

Regarding subjective symptom duration, we observed a wide range: between 1 day up to 19 years (10.7 ± 33.3 months, median 1 month). On this account, we focused on symptoms started within 12 months before presentation and compared stages accordingly. Here again, there was a wide range and no clear correlation between symptom duration and initial disease stage (Figure 2 lower row). However, patients in stage 3B reported about significantly longer symptom durations (4.5 ± 2.1 months, range 3–6 months, median 4.5 months) than patients in stage 2A (1.9 ± 1.5 months, range 3 weeks–6 months, median 1 month, *p* = 0.046).

### 3.4 Final visual acuity

**FIGURE 3:**
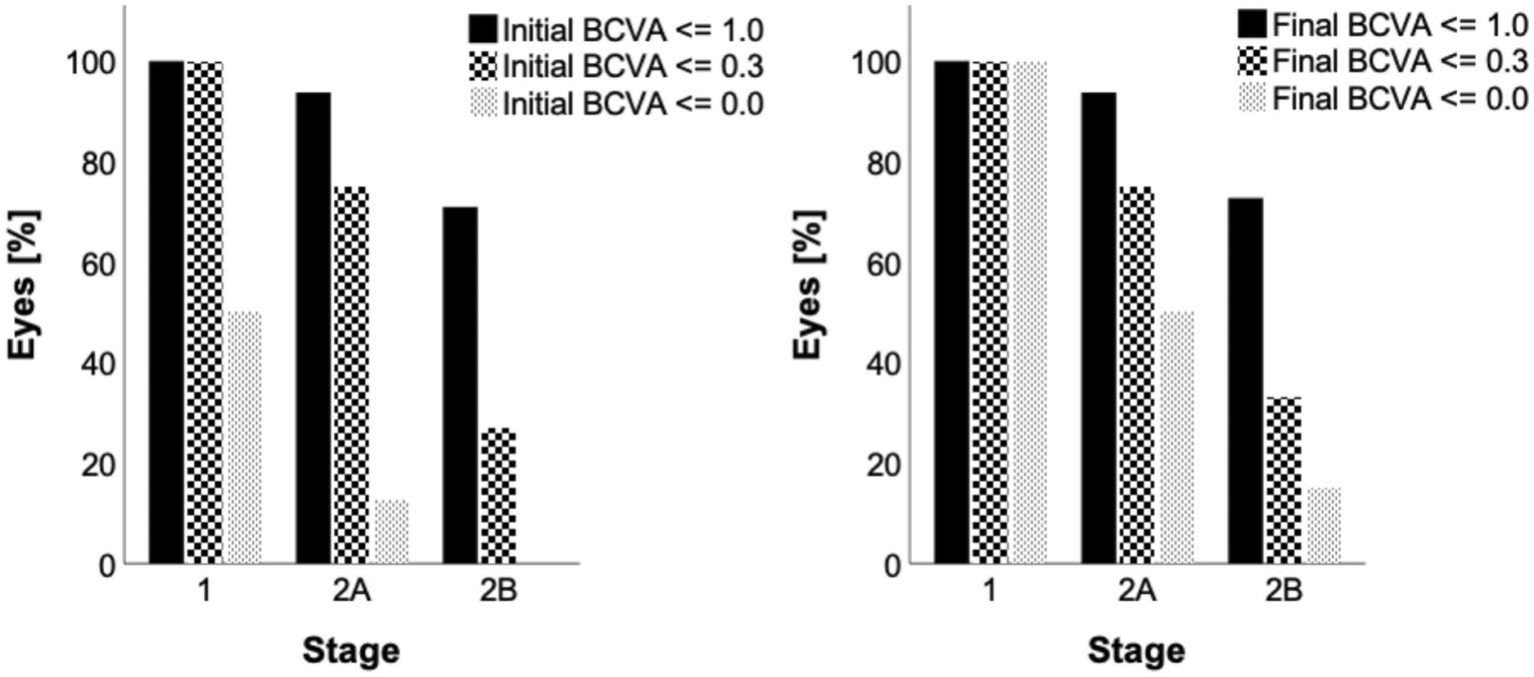
Percentage of eyes with an initial and final best corrected visual acuity (BCVA) equal or superior to a logarithm of minimal angle of resolution (logMAR) of 1.0, 0.3 and 0.0 with regard to stage 1, 2A and 2B. Significances were omitted to avoid overload of the figure.

At the final visit, BCVA presented better visual outcomes in early stages (1/2A/2B) and poorer visual acuities in late stages (3A1/3A2/3A2/4/5; Figure 1 right diagram). We focused on stages 1, 2A and 2B; comparing the proportion of eyes progressing to a predefined BCVA with respect to initial BCVA (Figure 3). Eyes in stage 1 started with good visual acuity; all eyes started at BCVA of ≤0.3 logMAR, half of these eyes had full BCVA (≤0.0 logMAR). At the final visit, all eyes of stage 1 attained full BCVA. Regarding stage 2A, 12.5% of cases started with full BCVA (2/16 eyes), which increased to 50.0% (8/16) after treatment. In contrast, none of the cases of stage 2B started with full BCVA at the initial visit. However, of these cases, 15.2% (5/33) attained full BCVA after treatment.

### 3.5 Stage 2B

**FIGURE 4:**
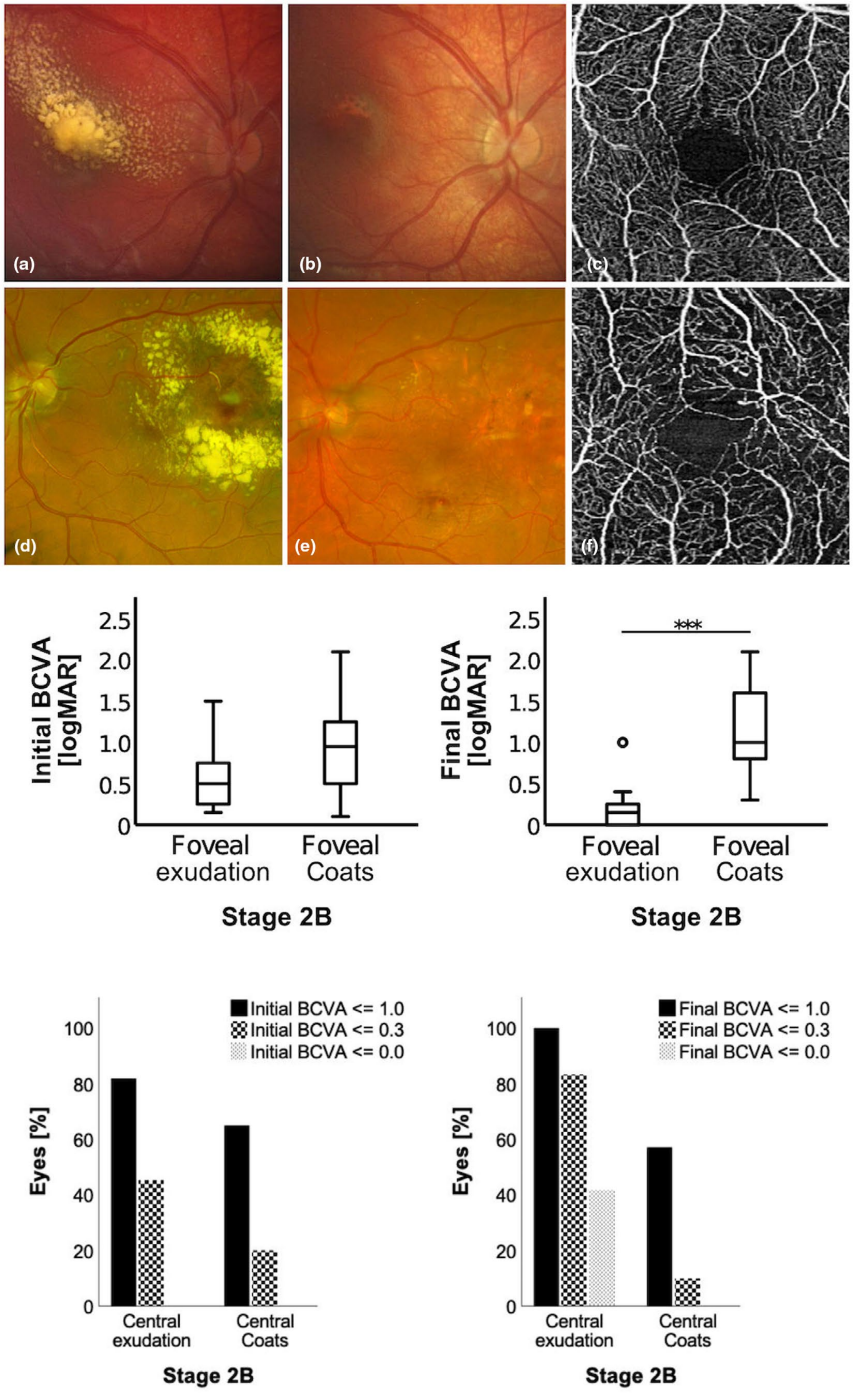
Upper row, development between two eyes with stage 2B, one with foveal exudation only (a–c) and one with foveal exudation and foveal Coats' typical vascular abnormalities (d–f). (a) stage 2B with foveal exudation only, presentation at initial visit; (b) presentation of the same eye at final visit after treatment with laser‐photocoagulation and cryocoagulation, best corrected visual acuity 1.0 logMAR; (c) optical coherence tomography angiography shows regular foveal avascular zone; (d) stage 2B with foveal exudation and foveal Coats' typical vascular abnormalities, presentation at initial visit; (e) presentation of the same eye at final visit after treatment with laser‐photocoagulation, best corrected visual acuity 0.7 logMAR; (f) optical coherence tomography angiography shows telangiectasia, capillary bed abnormalities, distorted foveal avascular zone and microaneurysms. Middle row, initial (left diagram) and final (right diagram) best corrected visual acuity (BCVA) given as logarithm of minimal angle of resolution (logMAR) with regard to the stage 2B divided into eyes with foveal exudation only versus additional foveal Coats' typical vascular abnormalities. Lower row, percentage of eyes with an initial (left diagram) and final (right diagram) best corrected visual acuity (BCVA) equal or superior to a logarithm of minimal angle of resolution (logMAR) of 1.0, 0.3 and 0.0 with regard to the stage 2B divided into eyes with foveal exudation only versus additional foveal Coats' typical vascular abnormalities. n.s., not significant; ***, *p* < 0.001.

Stage 2B comprises eyes with central macular exudation involving the fovea. This exudation can be a consequence of peripheral vascular abnormalities or derive from the exudation of additional foveal vascular abnormalities, such as telangiectasia, capillary bed abnormalities, distorted foveal avascular zone and microaneurysms. Both entities present with distinct clinical courses (Figure 4 upper row). While eyes with foveal exudation only obtained normal configuration of the macular and fovea when exudation is resorbed after adequate treatment of extrafoveal vascular lesions (Figure 4a–c), eyes with foveal Coats' typical vascular abnormalities and additional foveal exudation did not achieve complete regression of foveal pathologies independent of the treatment applied (Figure 4d–f). Therefore, we compared eyes with stage 2B and foveal exudation only (*n* = 12) to eyes with stage 2B and additional foveal Coats' typical vascular abnormalities (*n* = 21). No significant differences were found regarding age, duration of symptoms, initial IOP and follow‐up time (*p* > 0.05, respectively). While initial BCVA did not significantly differ between groups (0.58 ± 0.43 vs. 0.91 ± 0.56 logMAR; *p* = 0.096; Figure 4 middle row left), final BCVA was significantly better in eyes with foveal exudation only (0.20 ± 0.29 logMAR), compared to eyes with additional foveal vascular abnormalities (1.20 ± 0.55 logMAR; *p* < 0.001; Figure 4 middle row right). The proportion of eyes that were able to regain full BCVA (≤0.0 logMAR) within these two subgroups may illustrate this circumstance even more (Figure 4 bottom row): none of the eyes in stage 2B presented with full BCVA at the initial visit (≤0.0 logMAR; Figure 4 bottom row left). At the final visit, 41.7% (5/12) in stage 2B with foveal exudation only attained full BCVA, which stands in contrast to the observation that none of the cases in stage 2B with foveal Coats' typical vascular abnormalities (0.0%; 0/21) attained full BCVA (Figure 4 bottom row right). Accordingly, within stage 2B with foveal exudation only, the proportion of eyes with full BCVA increased from 75.0% (9/12) to 100% (12/12); and proportion of eyes with BCVA of 0.3 logMAR or better increased from 41.7% (5/12) to 83.3% (10/12) during treatment. In comparison, corresponding to this evaluation of visual acuities, the proportions of eyes with foveal Coats' associated vascular abnormalities decreased in both categories (Figure 4). However, we also found eyes with persistent macular scars after extensive treatment (e.g. cryotherapy) of extrafoveal vascular pathologies and subfoveal pseudodrusen from condensed exudates (8/77; 10.4%).

### 3.6 Stage‐dependent treatment

Within our cohort the following treatment options were applied in a stage‐dependent manner: laser‐photocoagulation (LAKO), cryocoagulation (CRYO), intravitreal injection (IVI) of anti‐VEGF drugs or steroids, pars plana vitrectomy (PPV), ruthenium brachytherapy (RU) in cases of secondary vasoproliferative retinal tumour (VPRT) and enucleation. As presented in Table S3, dominating procedures differed between stages. While eyes in stage 1 were only treated with LAKO (100.0%; 2/2), eyes in stage 2A and 2B were treated with LAKO (2A:62.3%; 10/16; 2B:87.9%; 29/33) and CRYO (2A:31.3%; 5/16; 2B:30.3%; 10/33). Additional IVI was applied in 12.5% in stage 2A (2/16) and in 30.3% in stage 2B (10/33). PPV was performed in 25.0% in stage 2A (4/16) and in 21.1% in stage 2B (7/33); RU was less frequent (2A:18.8%; 3/16; 2B:6.1%; 2/33).

This stands in contrast to stage 3A1, in which every eye received CRYO (100%; 9/9) and in 88.9% (8/9) additional LAKO. Within stage 3A1, the rate of cases requiring PPV was higher (44.4%; 4/9). Each eye in stage 3A2, 3B and 5 received PPV (100%, 3A2:3/3; 3B:5/5; 5:2/2). LAKO and CRYO were the second or third most frequent treatment options in stage 3A2 (66.7%; 2/3) and 3B (80%; 4/5). The enucleation rate was highest in stage 4 (57.1%; 4/7), followed by stage 5 (50%; 1/2).

Comparing stage 2B1 (foveal exudation only) with 2B2 (foveal Coats' typical vascular abnormalities), percentages of eyes receiving one of the applied interventions were similar, except for the number of IVI, which was higher in stage 2B1 (58.3%; 7/12) vs. 14.3%; 3/21 (Table S3).

Finally, number of interventions regarding initial stage was analysed (Table S4). Here, highest number of LAKO sessions was performed in stage 2B (4.8, range 1–25), most sessions of CRYO were performed in stage 3B (2.8, range 1–6) and 3A1 (mean 2.7, *range 1–7).

Additional IVI was mainly repeated in stage 2B (mean 3.1, range 1–9) and the highest number of PPV was performed in stage 3A2 (mean 3.7, range 1–5). If RU was necessary due to VPRT, it was applied only once per eye.

We analysed our data regarding eyes in Stage 2B treated with IVI versus eyes without intravitreal anti‐VEGF therapy. Almost one third of eyes (30.3%) within Stage 2B received at least one IVI. Comparing the visual acuity of eyes with (10/33) and without IVI (23/33) no significant differences were found for the final BCVA (0.62 ± 0.69 versus 0.93 ± 0.66; *p* = 0.115). Before 2010, no IVI was performed within our study cohort (first IVI was April 21, 2010). Thus, we analysed the subgroup Stage 2B, treated from 2010 until 2017 (*n* = 16). Ten eyes received at least one IVI (initial BCVA: 0.68 ± 0.44), whereas 6 eyes did not undergo IVI (initial BCVA: 0.69 ± 0.66; *p* = 0.481). The final BCVA within this subgroup did not significantly differ (0.62 ± 0.69 versus 0.48 ± 0.44; *p* = 0.336).

Within stage 2B, the mean number of LAKO, CRYO sessions and IVI was slightly higher in stage 2B2 (foveal Coats' typical vascular abnormalities) compared to stage 2B1 (foveal exudation only).

## 4 DISCUSSION

All stages of Coats' disease were represented within our study group (77 eyes; mean follow‐up 7 years). While stage 1 represents a very early stage with only telangiectasia and without any exudation, stage 5 (end stage) is characterized as a blind, non‐painful eye with total retinal detachment, often with cataract and phthisis bulbi. Here we provide extended long‐term follow‐up (7.2 years; median 6.3 years; range, 13 months–21.1 years) of patients with Coats' disease, who were assessed for long‐term development of BCVA, irrespective of treatment performed. In contrast, previous studies of Coats' disease with a sufficient number of patients had shorter follow‐up periods (2–4 years, often considerable shorter medians) with a minimum follow‐up of 6 months (Shields et al., 2001, 2019a, 2019b).

Most eyes of our cohort presented with stage 2B (>40%). This is exceptional, compared to data of Shields who assigned 6–14% of their patients to stage 2B (Shields et al., 2001, 2019a, 2019b). Stage 2B marks the transition from good (≤0.3 logMAR) to poor initial BCVA (≥1.0 logMAR) within our study. This correlates with early presentation of symptoms, which significantly differed between stages in our cohort. Patients with affection of the central retina, especially within stage 2B, reported complaints early and they most frequently stated loss of visual acuity. While the finally attained BCVA of stage 2B varies widely, the presence of foveal vascular abnormalities was identified as an important determinant of outcome. On this account, we here recommend a refinement of the well‐established classification system as published by Shields, suggesting a subdivision of stage 2B (telangiectasia and exudation with foveal exudation) into 2B1 (telangiectasia and exudation with foveal exudation) and 2B2 (telangiectasia and exudation with foveal exudation and foveal Coats' typical vascular abnormalities; Table 1).

**TABLE 1:** Refined classification of Coats' disease according to Shields.

Stage	Characteristics
1	Retinal telangiectasia only
2A	Telangiectasia and exudation with extrafoveal exudation
2B1	Telangiectasia and exudation with foveal exudation
2B2	Telangiectasia and exudation with foveal exudation and foveal Coats' typical vascular abnormalities
3A1	Exudative retinal detachment, subtotal detachment, extrafoveal
3A2	Exudative retinal detachment, subtotal detachment, foveal
3B	Exudative retinal detachment, total retinal detachment
4	Total retinal detachment and glaucoma
5	Advanced end‐stage disease (blind, non‐painful, phthisis)

This is based on significant differences of BCVA between subgroups at final follow‐up. A significantly superior final BCVA was present in patients with foveal exudation only, in contrast to eyes with Coats' typical vascular abnormalities in the foveal area.

While initial BCVA did not significantly differ between these groups (2B1/2B2), the reduction of final visual acuity might be the result of structural damages. This was present in eyes with macular vascular abnormalities and exudates. These results were furthermore confirmed by analyses of proportions of patients attaining a predefined BCVA (≤1.0, 0.3 or 0.0 logMAR) during follow‐up (Figure 4). While none of the eyes within stage 2B initially presented with full BCVA (≤0.0 logMAR), about half of the eyes in the group with foveal exudation only (stage 2B1) attained full BCVA at the final examination, compared to no eye with foveal Coats' typical vascular abnormalities (stage 2B2).

Furthermore, in previous studies of Shields et al., most patients presented with stage 3 (67–68% (Shields et al., 2001, 2019a, 2019b)), while within our cohort only 22% had stage 3 at the initial visit. These results suggest an earlier detection of vascular abnormalities potentially due to preventive ophthalmological examinations and a higher sensibility, which might also be supported by currently used advanced UWFI techniques for Coats' disease nowadays.

Stage 1, as a very early stage, was only found in two patients of our cohort (2.6%), that is in concordance with the findings of Shields et al. (2001, 2019a, 2019b). In general, there is no requirement for any intervention in Coats' disease stage 1. Thus, a possible reason for the limited number of patients could be seen in selection bias to our tertiary referral centre. In addition, telangiectasia that often present in the retinal periphery do not cause visual complaints, may be underdiagnosed and the estimated number of patients with stage 1 might be higher.

The advanced stages 4 and 5 represented a minority of patients in our study cohort, which is in accordance with the studies by Shields et al. (9% and 2.6% vs. 6–14% and 1–2%; Shields et al., 2001, 2019a, 2019b).

As expected, a stage‐dependent treatment regime was applied. Thereby, less invasive methods were used in early stages, as compared to advanced stages 4 and 5, whereas enucleations became necessary in a few cases. This is in accordance with the findings of Shields et al. (2001, 2019a, 2019b). Interestingly, the frequency of comparable interventions was found to be considerably higher within our cohort. In this respect, it remains unclear how interventions were performed in detail (e.g. number of LAKO‐/CRYO spots per session). Shields et al. did not include vitrectomy in advanced stages or brachytherapy in cases of secondary VRPT. On the other hand, we did only perform sub‐Tenon's corticosteroid injections to reduce inflammation postoperatively, but not as a separate treatment option.

Since the introduction of intravitreal anti‐VEGF therapy, adjuvant injections were also used to treat patients with Coats' disease. We investigated the course of BCVA between the proposed subgroups 2B1 (Telangiectasia and exudation with foveal exudation) and 2B2 (Telangiectasia and exudation with foveal exudation and foveal Coats' typical vascular abnormalities) from 2010 until 2017 (*n* = 16; 2B1: *n* = 9; 2b2: *n* = 7). Within stage 2B1 77.8% (7/9) received IVI, while in stage 2B2 42.9% (3/7) underwent IVI. Initial BCVA in 2B1 with IVI was 0.59 ± 0.46 versus without IVI 0.23 ± 0.11 (*p* = 0.164). Final BCVA in 2B1 with IVI was 0.27 ± 0.35 versus without IVI 0.0 ± 0.0 (*p* = 0.165). Within stage 2B2, the initial BCVA in 2B2 was not significantly different (1.0 ± 0.0 with IVI versus 0.93 ± 0.7 without IVI; *p* = 0.448). However, the final BCVA in 2B2 with IVI was 1.43 ± 0.59, while eyes without IVI in this stage had a better final BCVA of 0.73 ± 0.31 (*p* = 0.045). Therefore, we cannot assume that eyes treated with IVI end up with better BCVA than eyes without IVI. Nevertheless, within our subgroups stage 2B1 and 2B2, the initial BCVA of eyes treated with IVI tended to be worse compared to eyes without IVI. This might bias the results of final BCVA.

Current classification according to Shields is predominantly based on vascular pathologies including clinical appearance to indicate disease stages: yellowish exudation, macular oedema, retinal detachment, secondary glaucoma. Later published analyses always related to this classification system, which is well‐established today. Recently released studies on Coats' disease focused on detailed description of morphological parameters using modern multimodal imaging tools such as UWFI including UWFFA, SD‐OCT and OCT angiography (Brockmann et al., 2021; Hautz et al., 2017; Jung et al., 2018; Rabiolo et al., 2017; Schwartz et al., 2018; Stanga et al., 2019). Thereby, findings of Rabiolo et al., Jung et al. and Hautz et al. increase understanding of microstructural pathologies in affected eyes. Due to novelty of the currently used multimodal imaging tools, even though long‐term data are not available at this point of time; these techniques will improve our understanding of retinal vascular abnormalities and help to earlier detect Coats' disease within next decades. To the best of our knowledge, the current study is the first long‐term follow‐up investigation of Coats' disease including a detailed analysis of foveal morphology.

We are aware that our study has some limitations, mainly due to its retrospective nature over a period of 27 years, in which examination methods and technical standards have changed. In cases of very young patients, initial BCVA could not be obtained in each case. Furthermore, we do have a strongly selective cohort of patients at our tertiary referral university centre— predominantly consisting of complex and pretreated cases. After a good response to treatment, patients were sent back to their primary ophthalmologists in several cases; these patients are potentially lost to follow‐up. Difficult cases with insufficient treatment response and repeated needs for treatments frequently remained under observation within our university centre (negative selection bias).

Taken together, classification of Coats' disease by Shields et al. is a carefully selected and accurate system that shows correlation between disease stages and initial as well as final parameters such as visual acuity and need for interventions, which is also supported by our data.

To improve predictability of final BCVA, we propose to include subdivision of stage 2B into 2B1 and 2B2, taking into account absence or presence of foveal Coats' typical vascular abnormalities.

## FUNDING INFORMATION

None.

## CONFLICT OF INTEREST STATEMENT

No conflicting relationship exists for any author.

## Supporting information


Figure S1.

Table S1.

Table S2.

Table S3.

Table S4.
