# Supplementary figures and images for: Long‐term outcome of Coats' disease: Implications for the classification of foveal vascular pathologies
Source: Acta Ophthalmol. 2025 Jul 5;104(1):e104–11. doi: 10.1111/aos.17554 (PMC12803690; doi:10.1111/aos.17554)

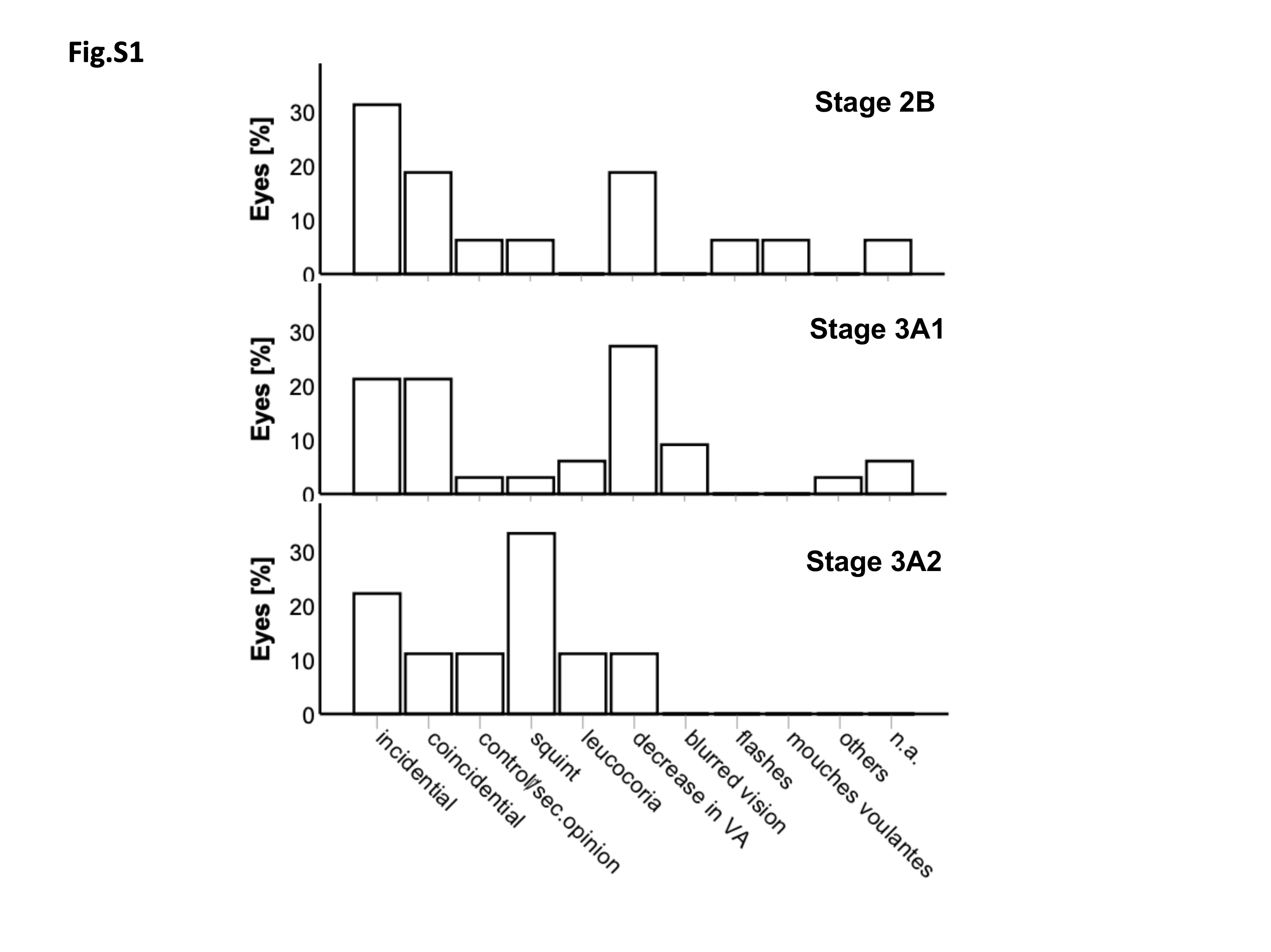

Supplement: Supplementary file 1 — Figure S1. [file AOS-104-e104-s003.tiff]
