# Supplementary material for: Long‐term outcome of Coats' disease: Implications for the classification of foveal vascular pathologies
Source: Acta Ophthalmol. 2025 Jul 5;104(1):e104–11. doi: 10.1111/aos.17554 (PMC12803690; doi:10.1111/aos.17554)
Supplement: Supplementary file 2 — Table S1. [file AOS-104-e104-s001.docx]

| **Tab.S1.** Visual acuity and intraocular pressure with regard to the initial stage | | | | | | | | | |
| --- | --- | --- | --- | --- | --- | --- | --- | --- | --- |
| **Parameter** | **Stage 1** | **Stage 2A** | **Stage 2B** | **Stage 3A1** | **Stage 3A2** | **Stage 3B** | **Stage 4** | **Stage 5** | **Total** |
| **Initial BCVA**  **(logMAR)** | 0.15 ± 0.21 (0.0 – 0.3, 0.2) | 0.27 ± 0.34 (-0.1 – 1.0, 0.2) | 0.79 ± 0.54 (-0.1 – 1.3, 0.7) | 1.68 ± 0.66; (0.7 – 2.1, 2.0) | 1.67 ± 0.25; (1.4 – 1.9, 1.7) | 2.27 ± 0.67; (1.7 – 3.0, 1.2) | 2.22 ± 0.74; (1.0 – 3.0, 2.1) | 2.10 ± 1.27; (1.2 – 3.0, 2.1) | 0.98 ± 0.83 (-1.0 – 3.0, 0.9) |
| **Final BCVA (logMAR)** | -0.05 ± 0.07 (-0.1 – 0.0, -0.1) | 0.22 ± 0.39 (-0.1 – 1.2, 0.1) | 0.84 ± 0.68 (0.0 – 2.1, 0.8) | 2.0 ± 0.88; (1.0 – 3.0, 2.0) | 1.77 ± 0.40; (1.3 – 2.0, 2.0) | 2.18 ± 0.62; (1.5 – 3.0, 2.1) | 2.72 ± 0.46; (2.0 – 3.0, 3.0) | 2.50 ± 0.71; (2.0 – 3.0, 2.5) | 1.14 ± 1.02 (-1.0 – 3.0, 1.0) |
| **Initial IOP (mmHg)** | 21.0 ± 0.0 (21 – 21, 21) | 13.5 ± 2.3 (10 – 16, 15) | 15.9 ± 3.4 (10 – 23, 15) | 13.8 ± 2.8 (11 – 18, 13) | 15.0 ± 0.0 (15 – 15, 15) | 17.0 ± 0.0 (17 – 17, 17) | 36.6 ± 16.7 (14 – 60, 39) | 9.5 ± 7.8 (4 – 15, 20) | 17.2 ± 9.0 (4 – 60, 15) |
| **Final IOP (mmHg)** | 16.0 ± 0.0 (16 – 16, 16) | 15.8 ± 2.1 (11 – 18, 17) | 15.1 ± 4.0 (5 – 25, 16) | 14.0 ± 3.0 (9 – 17, 15) | 15.3 ± 5.0 (10 – 20, 16) | 20.0 ± 1.7 (18 – 21, 21) | 21.0 ± 28.6 (3 – 54, 06) | 5.0 ± 0.0 (5 – 5, 5) | 15.6 ± 6.6 (3 – 54, 16) |
| **BCVA**, best-corrected visual acuity; **logMAR**, logarithm of the minimum angle of resolution; **IOP**, intraocular pressure; Results are given as mean value ± standard deviation (minimum – maximum, median). | | | | | | | | | |
