# Supplementary material for: Long‐term outcome of Coats' disease: Implications for the classification of foveal vascular pathologies
Source: Acta Ophthalmol. 2025 Jul 5;104(1):e104–11. doi: 10.1111/aos.17554 (PMC12803690; doi:10.1111/aos.17554)
Supplement: Supplementary file 3 — Table S2. [file AOS-104-e104-s004.docx]

| **Tab.S2.** P-values comparing stages with regard to initial best-corrected visual acuity | | | | | | | | |
| --- | --- | --- | --- | --- | --- | --- | --- | --- |
|  | **Stage 1** | **Stage 2A** | **Stage 2B** | **Stage 3A1** | **Stage 3A2** | **Stage 3B** | **Stage 4** | **Stage 5** |
| **Stag 1** | - | n.s. | n.s. | 0.038 | 0.006 | 0.025 | 0.010 | n.s. |
| **Stage 2A** | n.s. | - | < 0.001 | < 0.001 | < 0.001 | < 0.001 | < 0.001 | n.s. |
| **Stage 2B** | n.s. | < 0.001 | - | 0.005 | 0.009 | < 0.001 | < 0.001 | 0.004 |
| **Stage 3A1** | 0.038 | < 0.001 | 0.005 | - | n.s. | n.s. | n.s. | n.s. |
| **Stage 3A2** | 0.006 | < 0.001 | 0.009 | n.s. | - | n.s. | n.s. | n.s. |
| **Stage 3B** | 0.025 | < 0.001 | < 0.001 | n.s. | n.s. | - | n.s. | n.s. |
| **Stage 4** | 0.010 | < 0.001 | < 0.001 | n.s. | n.s. | n.s. | - | n.s. |
| **Stage 5** | n.s. | n.s. | 0.004 | n.s. | n.s. | n.s. | n.s. | - |
| Results are given as mean value ± standard deviation (minimum – maximum, median); **n.s.**, not significant. | | | | | | | | |
