# Supplementary material for: Long‐term outcome of Coats' disease: Implications for the classification of foveal vascular pathologies
Source: Acta Ophthalmol. 2025 Jul 5;104(1):e104–11. doi: 10.1111/aos.17554 (PMC12803690; doi:10.1111/aos.17554)
Supplement: Supplementary file 4 — Table S3. [file AOS-104-e104-s005.docx]

| **Tab.S3.** Percentage of eyes receiving treatment for Coats’ disease with regard to the initial stage | | | | | | |
| --- | --- | --- | --- | --- | --- | --- |
| **Stage** | **LAKO** | **Cryo** | **IVI** | **PPV** | **Ru** | **Enu** |
| **1** | 100 (2/2) | - | - | - | - | - |
| **2A** | 62.3 (10/16) | 31.3 (5/16) | 12.5 (2/16) | 25.0 (4/16) | 18.8 (3/16) | - |
| **2B** | 87.9 (29/33) | 30.3 (10/33) | 30.3 (10/33) | 21.1 (7/33) | 6.1 (2/33) | - |
| **3A1** | 88.9 (8/9) | 100.0 (9/9) | 11.1 (1/9) | 44.4 (4/9) | - | - |
| **3A2** | 66.7 (2/3) | 33.3 (1/3) | - | 100.0 (3/3) | - | - |
| **3B** | 80.0 (4/5) | 80.0 (4/5) | 20.0 (1/5) | 100.0 (5/5) | - | - |
| **4** | 14.3 (1/7) | 28.6 (2/7) | - | 28.6 (2/7) | 14.3 (1/7) | 57.1 (4/7) |
| **5** | - | - | - | 100.0 (2/2) | - | 50.0 (1/2) |
| **LAKO**, photolasercoagulation; **Cryo**, cryocoagulation; **IVI**, intravitreal injection; **PPV**, pars plana vitrectomy; **Ru**, ruthenium brachytherapy due to secondary vasoproliferative retinale tumor; **Enu**, enucleation; total number of eyes are given in brackets. | | | | | | |
