# Supplementary material for: Long‐term outcome of Coats' disease: Implications for the classification of foveal vascular pathologies
Source: Acta Ophthalmol. 2025 Jul 5;104(1):e104–11. doi: 10.1111/aos.17554 (PMC12803690; doi:10.1111/aos.17554)
Supplement: Supplementary file 5 — Table S4. [file AOS-104-e104-s002.docx]

| **Tab.S4.** Number of interventions of eyes receiving treatment for Coats’ disease with regard to the initial stage | | | | | | |
| --- | --- | --- | --- | --- | --- | --- |
| **Stage** | **LAKO** | **Cryo** | **IVI** | **PPV** | **Ru** |  |
| **1** | 2.0 (2-2) | - | - | - | - |  |
| **2A** | 1.9 (1-3) | 1.4 (1-3) | 1.5 (1-2) | 0 (1-1) | 1.0 (1-1) |  |
| **2B** | **4.8 (1-25)** | 2.0 (1-3) | **3.1 (1-9)** | 2.6 (1-4) | 1.0 (1-1) |  |
| **3A1** | 2.8 (1-8) | **2.7 (1-7)** | 2.0 (2-2) | 1.8 (1-4) | - |  |
| **3A2** | 3.0 (3-3) | 1.0 (1-1) | - | **3.7 (1-5)** | - |  |
| **3B** | 3.3 (2-5) | **2.8 (1-6)** | 1.0 (1-1) | 1.8 (1-3) | - |  |
| **4** | 2.0 (2-2) | 2.0 (1-3) | - | 2.0 (1-3) | 1.0 (1-1) |  |
| **5** | - | - | - | 1.0 (1-1) | - |  |
| Numbers are given as mean (minimum – maximum). **LAKO**, photolasercoagulation; Cryo, cryocoagulation; **IVI**, intravitreal injection; **PPV**, pars plana vitrectomy; **Ru**, ruthenium brachytherapy due to secondary vasoproliferative retinale tumor. Highest values per group are written in bold letters. | | | | | | |
